# Impulse oscillometry in patients with pulmonary arterial hypertension: an exploratory study

**DOI:** 10.1016/j.clinsp.2023.100313

**Published:** 2024-03-14

**Authors:** Helena Jannuzzi Villas Bôas, Ilma Aparecida Paschoal, Mônica Corso Pereira

**Affiliations:** Departamento de Medicina Interna, Universidade de Campinas (UNICAMP), Campinas, SP, Brazil

**Keywords:** Pulmonary arterial hypertension, Pulmonary hypertension, Impulse oscillometry, Spirometry, Lung function

## Abstract

**Introduction:**

Studies suggest peripheral airway abnormalities in Pulmonary Arterial Hypertension (PAH). Impulse Oscillometry (IOS) is a noninvasive and sensitive technique for assessing the small airways. It evaluates the impedance of the respiratory system ‒ Resistance (R) and reactance (X) ‒ to a pulse of sound waves sent to the lungs, in a range of frequencies (5‒20 Hz).

**Method:**

Resistance variables: R5, R20, R5-R20 and reactance variables: AX (reactance area) and Fres (resonance frequency). The aim is to evaluate R and X in patients with idiopathic PAH (IPAH) and to investigate whether there is a correlation between IOS and spirometry.

**Results:**

Thirteen IPAH patients and 11 healthy subjects matched for sex and age underwent IOS and spirometry. IPAH patients had lower FVC and FEV_1_ values (*p* < 0.001), VEF_1_/CVF (*p* = 0.049) and FEF 25-75 (*p* = 0.006) than healthy patients. At IOS, IPAH patients showed lower tidal volumes and higher AX (*p* < 0.05) compared to healthy individuals, and 53.8 of patients had R5-R20 values ≥ 0.07 kPa/L/s. Correlation analysis: X5, AX, R5-R20 and Fres showed moderate correlation with FVC (*p* = 0.036 *r* = 0.585, *p* = 0.001 *r* = -0.687, *p* = 0.005 *r* = -0.726 and *p* = 0.027 *r* = -0.610); Fres (*p* = 0.012 *r* = -0.669) and AX (*p* = 0.006 *r* = -0.711) correlated with FEV_1_; [R5 and R20, (R5-R20)] also correlated with FEV_1_ (*p* < 0.001 *r* = -0.573, *p* = 0.020 *r* = -0.634 and *p* = 0.010 *r* = -0.683, respectively) in the IPAH group. There were also moderate correlations of FEF 25–75 % with Z5 (*p* = 0.041), R5 (*p* = 0.018), Fres (*p* = 0.043) and AX (*p* = 0.023).

**Discussion:**

Patients showed changes suggestive of increased resistance and reactance in the IOS compared to healthy individuals, and the IOS findings showed a good correlation with spirometry variables.

## Highlights

• Impulse Oscillometry (IOS) can be used to analyze lung resistance and reactance in patients with PAH.
• Patients with PAH show increased resistance and pulmonary reactance compared to healthy individuals.
• The IOS findings showed a good correlation with the spirometric variables.

## Introduction

Pulmonary Arterial Hypertension (PAH) is a progressive disease for which there is yet no curative treatment. It is characterized by the presence of remodeling of small pulmonary blood vessels, causing an increase in vascular resistance and an increase in pulmonary arterial pressure. The diagnosis of PAH is confirmed by hemodynamic findings such as evidence of elevated mean pulmonary arterial pressure (> 20 mmHg) with pulmonary capillary occlusion pressure below 15 mmHg and an increase in pulmonary vascular resistance above 2 Woods.^1^ The absence of concomitant disease or other etiology for PAH characterizes the idiopathic form (IPAH).^2^

Dyspnea and limitation of performance are the main symptoms of patients with PAH. Hemodynamic abnormalities caused by increased vascular resistance and increased pulmonary arterial pressure leading to pressure overload in the right ventricles are the mechanisms that cause dyspnea and performance limitation and have been the best studied to date.^3^

However, there is increasing evidence that ventilatory mechanisms are important as factors contributing to dyspnea in these patients.^4^ The presence of airway changes in patients with PAH has been documented in experimental studies with animal models^5^ and in some studies with human populations.^6^ Some authors suggest that the peripheral airways are the main site of obstruction in patients with PAH.^7^ Because spirometry is limited in assessing small airway obstruction, other techniques for assessing this lung region can be tested to detect an increase in airway resistance in these patients.^8^

Impulse Oscillometry (IOS) is a noninvasive technique that uses sound waves to evaluate airway impedance and its components, resistance, and reactance.^9^ Resistance is the in-phase component of impedance and provides information about the energy required to propagate the pressure wave through the airways, including bronchi, bronchioles, and distension of the lung parenchyma. Reactance is an out-of-phase component of impedance and reflects the capacitance and inertial properties of the airway. It can be understood as a rebound resistance, or echo, providing information about the distensibility of the airways and air spaces. Reactance includes two components, (a) Inertance, which reflects the inertial force of the moving column of air, and (b) Capacitance (or elastance), which reflects the elastic properties of the lung, and can be understood here as related to energy storage: the lung distended during inspiration stores energy to return to the resting position during expiration.^10^, ^11^, ^12^, ^13^

The information obtained through the evaluation of these lung properties (resistance and reactance) contributes to the diagnosis and monitoring of lung function. The interpretation of IOS test results can be classified as normal, central obstruction, peripheral obstruction, and pulmonary restriction.^10^

The two main advantages of this technique are: (a) It is easy to perform and requires little patient cooperation; (b) It allows measurement of pulmonary resistance and reactance along the tracheobronchial tree, providing important information about the homogeneity of ventilation in the peripheral lung regions.^9^^,^^14^

Although it has already been used in the evaluation of asthma, COPD and interstitial diseases,^9^ there are not many studies using IOS in patients with PAH. Within this context, the objective of this study is to assess airway resistance and reactance in patients with PAH using IOS, and to investigate whether there is a correlation of IOS findings with spirometry variables.

### Methods and casuistic

This was an exploratory cross-sectional study conducted at a public university hospital in the Campinas region of São Paulo state, Brazil. It was a convenience sample of patients recruited from January to March 2019 and October 2019 to February 2020 from the Pulmonary Hypertension Outpatient Clinic of the University of Campinas ‒ UNICAMP. The project was approved by the Institutional Ethics Committee, Opinion nº 4.756.549, CAAE: 95282918.4.0000.5404, and all study participants signed an informed consent form.

Forty-one patients diagnosed with IPAH, aged 18 years or older, and with a dyspnea functional class between I, II, and III (New York Heart Association) were recruited from the UNICAMP outpatient pulmonology database.^15^ Individuals using continuous oxygen at home, cognitively unable to perform the proposed tests, and current smokers were not included in the study. Healthy subjects, volunteers without respiratory or cardiac disease, and nonsmokers were matched for sex and age. After the subjects were admitted and in agreement with them, a day was scheduled to perform impulse oscillometry and spirometry, both of which were performed by the researcher in a single visit to the hospital.

All participants underwent clinical examination before the start of the tests. Anthropometric data (weight and height), O_2_ saturation, and vital signs at rest were collected. Data on diagnosis and medications taken were collected with the patient or from the medical record.

The tests were always performed in the same order (spirometry after IOS). The device used to perform the lung tests was the Master Screen IOS (Erich Jaeger, Germany®) of the LAFIP ‒ CIPED laboratory of UNICAMP, which was calibrated with a three-liter syringe before each data collection period.

To perform the IOS, three tests considered valid were performed, the best of which was selected for further analysis. Each maneuver lasted 30 s so resistance values were averaged at frequencies of 5 and 20 Hz. The subject tested was instructed to remain in a seated position with feet parallel to the floor, spine straight and leaning against a chair, knees at a 90° angle, hands on cheeks applying light pressure to minimize loss of oscillatory pressure. The subject was instructed to use a nose clip, connect the mouth to the mouthpiece of the device, and breathe spontaneously. The variables measured were: impedance (Z), resistance variables (resistance at 5 Hz [R5], resistance index of the large and small airways; resistance at 20 Hz [R20], resistance index of the large airways; the difference between R5-R20, an indicator of peripheral airway function), and reactance variables (reactance at 5 Hz [X5], an indicator of the capacitive reactance of the peripheral airways; resonant frequency [Fres], the intermediate frequency at which the reactance is zero; and reactance area [AX], representing the total reactance at all frequencies between 5 Hz and Fres).^16^, ^17^, ^18^, ^19^

Spirometry was performed according to the American Thoracic Society guidelines.^20^ During the test, at least three acceptable maneuvers and two reproducible maneuvers were performed to ensure that there were no errors and, most importantly, no lack of understanding on the part of the patient. The following parameters were assessed: FVC (volume of air that can be exhaled from the lungs during a forced maneuver after maximal inspiration without a time limit) and FEV_1_ (volume exhaled in the first second of FVC).

To describe the individuals of each group, a descriptive analysis was used as mean, median, and standard deviation. The Mann-Whitney test was used to analyze the variations of the variables between groups. The Chi-Square test was used to compare proportions. Spearman's correlation coefficient was used for correlation analysis (SAS system for Windows-Statistic Analysis System-9.4). The significance level adopted for the statistical tests was 5 %.

## Results

Among the 41 patients diagnosed with IPAH identified, 12 did not meet the inclusion criteria, with five no contact made (after three attempts), eight patients refused to participate in the research and three patients died. Thus, the authors included and evaluated 13 patients in Idiopathic Pulmonary Arterial Hypertension Group (IPAHG). Eleven healthy individuals were included and evaluated in the Control Group (CG), and the demographic data are shown in Table 1. As expected, there was no significant difference in the comparison between the groups regarding demographic variables. A sedentary lifestyle, defined by the World Health Organization (WHO), 2020^21^ was identified in six individuals from each group, with a similar frequency between the two groups (*p* = 0.6820).

**Table 1:** Baseline characteristics of the analyzed individuals.

	IPAHG (n = 13)	CG (n = 11)	p-value
Female sex	12 (92.3)	9 (81.8)	0.576^c^
Age years	44.85 ± 12.82	37.91 ± 14.18	0.223^a^
Body mass index, kg/m^2^	25.11 ± 5.59	23.38 ± 3.23	0.602^a^
Functional class (I/II/III)	I - 3 (23.1)		
	II - 7 (53.8)	‒	‒
	III - 3 (23.1)		
Systemic arterial hypertension	1 (7.6)	1 (9)	‒
Sedentary lifestyle	6 (46.2)	6 (54.5)	0.682^b^
Vital signs			
Systolic blood pressure	102.31 ± 12.35	108.18 ± 11.68	0.151^a^
Diastolic blood pressure	66.92 ± 7.51	71.82 ± 13.28	0.439^a^
Heart rate	74.46 ± 14.09	67.09 ± 10.67	0.385^a^
Respiratory frequency	18.62 ± 3.80	16.18 ± 2.99	0.064^a^
Peripheral oxygen saturation	95.31 ± 2.21	96.91 ± 1.14	0.060^a^

Data expressed in number and frequency, or in mean and standard deviation. Based on the Mann-Whitney test^a^, Chi-square^b^ and Fisher's exact test^c^.

Functional class (NYHA) data (from the IPAHG) and vital signs (of all participants) are shown in Table 1. There was no difference in the comparison between the groups.

Table 2 shows the IOS and spirometry variables (values before bronchodilation) and the results of the comparison between the two groups. On spirometry, patients had lower FVC, FEV_1,_ and FEF 25–75 values compared with healthy subjects. At IOS, patients had lower tidal volume and greater reactance area than controls. Seven of the 13 patients with IPAH analyzed here had values of R5-R20 ≥ 0.07 kPa/L/s. Some authors have used the difference between (R5-R20) to examine the prevalence of small airway dysfunction, which is considered to occur when R5-R20 ≥ 0.07 kPa/L/s is detected.^9^^,^^14^

**Table 2:** Comparisons between oscillometry and spirometry variables between groups.

		IPAHG (n = 13)	CG (n = 11)	p-value
IOS	TV (L)	0.61 ± 0.27	1.07 ± 0.38	0.002
	Z5 [kPa/(L/s)]	0.43 ± 0.14	0.38 ± 0.09	0.417
	R5 [kPa/(L/s)]	0.40 ± 0.12	0.36 ± 0.09	0.542
	R20 [kPa/(L/s)]	0.32 ± 0.08	0.33 ± 0.08	0.816
	R5-R20[kPa/(L/s)]	0.08 ± 0.08	0.03 ± 0.02	0.063
	R5-R20 ≥0.07 kPa/(L/s)	7 (53.84)	0 (0)	‒
	X5 [kPa/(L/s)]	-0.16 ± 0.09	-0.12 ± 0.04	0.123
	Fres (L/s)	16.50 ± 7.14	11.67 ± 2.25	0.093
SPIROMETRY	AX (kPa/L)	0.97 ± 1.11	0.33 ± 0.16	0.034
	FVC (L)	3.01 ± 0.66	4.11 ± 0.69	0.001
	FVC%	86.65 ± 13.60	106.80 ± 8.12	0.001
	FEV_1_ (L)	2.28 ± 0.61	3.35 ± 0.79	0.001
	FEV_1_%	79.48 ± 15.06	104.55 ± 11.62	0
	FEF 25–75 %	61.69 ± 25.25	97.81 ± 27,20	0.006

TV, Tidal Volume; Z5, Impedance at 5 Hz; R5, Resistance at 5 Hz; R20, Resistance at 20 Hz; X5, Reactance at 5 Hz; Fres, Resonant frequency; AX, Reactance area; (R5-R20), difference between R5 and R20; FVC, Forced Vital Capacity; FEV_1_, Forced expiratory volume in 1s. Data expressed as mean and standard deviation. Based on the Mann-Whitney test (p ≤ 0.05).

Correlation analysis was performed between the IOS variables (TV, Z5, R5, R20, R5-R20, X5, Fres, AX) with the spirometry variables FVC, FEV_1,_ and FEF 25–75 % in absolute values and percent predicted (Table 3). Except for Tidal Volume (TV), all IOS variables correlated with spirometry. For resistance variables, there was a moderate correlation of several variables with FVC and FEV_1_. The authors emphasize that strong correlations between R5-R20 with FVC and FEV_1_ were observed in IPAHG. Strong correlations were observed between Fres and AX with FVC and FEV_1_ in the reactance variables, exclusively in the patient group. There were also moderate correlations of FEF 25–75 % with Z5, R5, Fres, and AX. It is also worth mentioning that in the group of healthy individuals, the variable R5-R20 obtained a negative correlation with FVC (in L) and Fres correlated inversely with VEF_1_ and FEF 25–75 %. An important finding worth studying with a larger number of patients.

**Table 3:** Correlation between IOS variables with and Spirometry.

Variables		IPAHG					CG				
		FVC (L)	FVC (%)	FEV_1_ (L)	FEV_1_ (%)	FEF 25-75 (%)	FVC (L)	FVC (%)	FEV_1_ (L)	FEV_1_ (%)	FEF 25-75 (%)
TV (L)	ρ correlation	-0.080	-0.325	-0.419	-0.330	-0.515	0.232	-0.378	0.150	-0.173	0.027
	p-value	0.796	0.279	0.154	0.271	0.072	0.492	0.252	0.659	0.611	0.936
Z5 (kPa/(L/s)	ρ correlation	-0.421	-0.534	-0.526	-0.752	-0.572	-0.400	-0.445	-0.382	-0.418	-0.273
	p-value	0.151	0.060	0.065	0.003	0.041	0.223	0.170	0.247	0.201	0.417
R5 (kPa/(L/s)	ρ correlation	-0.448	-0.558	-0.573	-0.818	-0.642	-0.402	-0.429	-0.384	-0.420	-0.296
	p-value	0.125	0.048	0.041	0.001	0.018	0.221	0.188	0.244	0.198	0.375
R20 (kPa/(L/s)	ρ correlation	-0.110	-0.199	-0.241	-0.634	-0.438	-0.202	-0.312	-0.202	-0.257	-0.174
	p-value	0.720	0.515	0.428	0.020	0.135	0.552	0.350	0.552	0.446	0.608
R5- R20 (kPa/(L/s)	ρ correlation	-0.726	-0.679	-0.683	-0.510	-0.517	-0.684	-0.244	-0.553	-0.384	-0.323
	p-value	0.005	0.011	0.010	0.075	0.071	0.020	0.470	0.078	0.243	0.332
X5 (kPa/(L/s)	ρ correlation	0.585	0.330	0.405	0.313	0.304	0.511	0.507	0.466	0.479	0.247
	p-value	0.036	0.271	0.170	0.297	0.312	0.108	0.112	0.149	0.136	0.465
Fres (l/s)	ρ correlation	-0.610	-0.560	-0.669	-0.610	-0.567	-0.533	-0.178	-0.601	-0.569	-0.752
	p-value	0.027	0.046	0.012	0.027	0.043	0.091	0.601	0.050	0.067	0.008
AX (kPa/L)	ρ correlation	-0.687	-0.516	-0.711	-0.593	-0.622	-0.542	-0.351	-0.524	-0.547	-0.374
	p-value	0.010	0.071	0.007	0.033	0.023	0.085	0.290	0.098	0.082	0.258

The correlation coefficient (ρ) can range from -1 (indicating a strong negative correlation between the two variables, i.e., when one grows the other decreases) to 1 (indicating a strong positive correlation between the two variables). When ρ is close to 0 it is concluded that there is no linear correlation between the two variables. Spearman correlation coefficient. Considered statistical significance when p≤0.05.

## Discussion

The present study showed that by evaluating the IOS findings, small airway dysfunction, expressed by the presence of R5-R20 ≥ 0.07,^9^^,^^14^ was present in 53.8 % of patients with IPAH. In addition, reactance area (AX), another parameter indicative of small airway impairment, was significantly increased compared with healthy subjects. In support of these findings, both variables (R5-R20 and AX) showed a moderate inverse correlation with the spirometry variables FVC and FEV_1_. Fres showed similar results in correlation analysis with FVC and FEV_1_. It is also worth noting that in the group of healthy subjects, only the variable R5-R20 showed a negative correlation with FVC (in L).

In one of the few studies that investigated IOS in patients with pulmonary hypertension, an increase in AX, Fres, and R5-R20 was also found in patients with PH compared with healthy individuals, a similar result that was found here.^8^ According to Komarow et al., the measurement of AX is sensitive to the assessment of peripheral airway function and is related to resistance at this site.^22^ Bonifazi et al^9^ studied the correlation between IOS variables and spirometry in a sample of patients with systemic sclerosis and found similar results to ours, with evidence of an inverse correlation between R5-R20 and AX with FEV_1_ in % of prediction (*r* = -0.3, *p* = 0.002 and *r* = -0.29, *p* = 0.005) and with FVC in % of prediction (*r* = -0.3, *p* = 0.002 and *r* = -0.29, *p* = 0.005).

Airway dysfunction is a phenomenon that has been described in some patients with PAH^3^ and its mechanisms are not fully understood. Some factors are thought to play a role, such as coexistence and competition for space between small hypertrophied vessels and distal airways in the interstitial space,^23^ wall thickening and/or the presence of a mucus plug in the small airways, possible airway hyperreactivity, and the interaction of some other factors, such as airway obstruction due to mechanical forces triggered by dilatation of the precapillary pulmonary arteries acting on the small inflamed airways.^1^^,^^24^^,^^25^

The patients studied here showed a decrease in tidal volume by IOS and a decrease in FVC, FEV_1,_ and FEF 25–75 on spirometry compared with the healthy group. The finding of decreased FVC is the most common finding on spirometry in patients with PAH, and several factors must contribute to this.^26^ Meyer and colleagues reported signs of peripheral airway obstruction defined by concomitant decreases in FVC and FEV_1_/FVC, as well as increased residual volume and increased ratio of residual volume to total lung capacity.^3^ These authors point to the possibility of premature airway obstruction leading to a reduction in FVC due to impaired elastic recoil of the lungs. de Almeida et al.^26^ in a study of patients with IPAH in which echocardiographic parameters during exercise were evaluated in comparison with spirometry and volumetric capnography, found a reduction in FVC and FEV_1_ (measured in Liters - L) even with a normal FEV_1_/FVC ratio, in addition to the fact that 42.9 % of patients had an FVC below the lower limit of normality, in contrast to none of the controls with this result. The present results are consistent with those of these studies and support the reduction of FVC as a functional characteristic of patients with PAH.

This is an exploratory study to investigate findings in respiratory impedance of patients with PAH, using IOS. The lack of reference values for the IOS variables limits the interpretation of the results of the present study. However, the pattern found was significantly different from that of healthy subjects, suggesting that the test may be useful for assessing and understanding the small airway dysfunction that occurs in some patients with PAH. This was a single-center study with a limited number of patients, so it may be important to conduct further studies with a larger number of patients for further analysis to expand the understanding of the role of IOS in assessing small airway function in patients with PAH.

## Conclusion

This exploratory study showed increased airway resistance and reactance (components of impedance) in patients with PAH compared with healthy individuals of the same age and sex. This finding can be related to distal small airway obstruction in IPAH subjects as another component of the pathogenesis of dyspnea and hypoxia in IPAH patients.

The results showed a good negative correlation with the variables of spirometry, an examination that is of limited use for the assessment of the small airways, with IOS.

It is important to highlight that, as this is a pilot study, the results obtained in the studied population cannot be generalized to all patients with IPAH. Further studies should be carried out to test whether the findings reported here are reproducible in other patient populations with PAH.

### Limitations of the study

This is an exploratory study to investigate findings in the respiratory impedance of patients with PAH, using the IOS. The lack of reference values for the IOS variables limits the interpretation of the findings of this study. However, the pattern found was clearly different from that found in healthy individuals, suggesting that the test may be useful in the evaluation and understanding of the small airway dysfunction that occurs in some patients with PAH. It was a single-center study, with a limited number of patients, so it may be important to conduct further studies with a larger number of patients for more analysis in order to broaden the understanding of the role of IOS in the assessment of small airway function in patients with PAH.

### Current knowledge

Dyspnea and limitation of performance are the main symptoms of patients with PAH. There is increasing evidence that ventilatory mechanisms are important factors contributing to dyspnea in these patients. Some authors suggest that the peripheral airways are the main site of obstruction in patients with PAH. Spirometry is limited in assessing small obstructions. IOS assesses this lung region and can be tested to detect an increase in airway resistance in these patients.

### What this paper contributes to our knowledge

IOS was a useful test, used to assess and understand small airway dysfunction in IPAH patients, even when patients had a normal spirometry test. Pulmonary function tests (spirometry and IOS) had moderate to strong correlations between their variables.

## Declaration of Competing Interest

The authors declare no conflicts of interest.

The present work was carried out with the support of the Coordination for the Improvement of Higher Education Personnel ‒ Brazil (CAPES) ‒ funding code 001.
